# Sustaining non-profit organizations: Strategic, managerial and financial perspectives

**DOI:** 10.1371/journal.pone.0339885

**Published:** 2026-01-02

**Authors:** Yara Harb, Lara Khabbaz, Marwan Azouri, Sandra Estephan

**Affiliations:** 1 Department of Management and Marketing, Notre Dame University-Louaize, Zouk Mosbeh, Lebanon; 2 Department of Marketing, Lebanese American University, Beirut, Lebanon; 3 Department of Management and Marketing, Notre Dame University-Louaize, Zouk Mosbeh, Lebanon; Paris School of Business, FRANCE

## Abstract

**Purpose:**

This study explores the complex challenges that Non-Governmental Organizations (NGOs) face. The research highlights the impact of strategic planning, managerial capabilities and financial management on NGOs sustainability. Sustainability is defined to mean durability and continuity of the organization.

**Design/methodology/approach:**

Data were collected from 75 NGOs using a survey to examine the roles of strategic planning, financial management, and managerial skills in supporting NGO sustainability.

**Findings:**

Contrary to initial assumptions, the findings reveal that while strategic planning and strong managerial capabilities are crucial for long-term sustainability, financial management does not show a direct impact. The study suggests that NGOs should focus on developing comprehensive strategic plans and enhancing their managerial capacities to effectively navigate complex environments and achieve their goals.

**Originality:**

This research contributes to the conversation on NGO management by highlighting the important intersection of resource-based competencies and stakeholder engagement in building resilient and successful organizations.

## Introduction

Non-governmental organizations (NGOs) play a critical role in addressing various social, economic, and environmental issues, especially in regions where governmental support is limited [1]. These organizations are vital in tackling inequities in service provision, advocating for human rights, and also promoting sustainable development [2]. The NGO community focuses on healthcare, education, economic alleviation, environmental preservation, and human rights, reflecting the multifaceted nature of challenges that NGOs face and the exceptional responses they provide [3]. The role of NGOs in delivering emergency humanitarian aid, shelter, and assistance during times of crisis is well documented, demonstrating their significance in national resilience [4]. Therefore, NGOs have been strongly associated with sustaining social welfare [5].

Sustainability, in the context of NGOs, is described as the long-term survivability and continuity of organizations [6]. The sustainability of NGOs is measured by its ability to endure and continue serving its constituencies while meeting its commitments to the community it serves. This typically happens when NGOs secure a steady stream of social, financial, and human resources [5]. Previous studies have highlighted various factors contributing to NGO sustainability. For instance, [7] emphasize the importance of responsible leadership and governance structures in achieving long-term sustainability for NGOs. [8] review the strategies used by NGOs to reduce vulnerability to poverty, discussing how effective programming and resource management contribute to sustainability. [1] explores the role of leadership in NGO sustainability, emphasizing the need for leaders to build inspiring visions and map out strategic directions to ensure long-term success.

To identify factors contributing to NGOs’ sustainability, three virtual focus group discussions were conducted, each involving 8–10 NGO representatives who later completed a survey. This method offered rich, contextual insights into NGO operations that surveys alone could not capture [9] enhancing the study’s validity [10]. A purposive sampling strategy ensured diversity in organizational type and stage [11]. With participants’ consent, sessions were recorded, transcribed, and analyzed using thematic analysis [12], leading to three key themes: strategic planning, financial management, and managerial effectiveness. Investigator triangulation was used to improve reliability [13], while member checking and reflexive journaling ensured credibility and reduced researcher bias [14,15]. Integrating these findings with quantitative data provided a comprehensive understanding of the enablers of NGO sustainability.

Despite the recognized importance of NGOs and their capabilities, their journey in striving towards continuity is still unclear. While NGOs rely heavily on donor funding, the impact of their internal capabilities, strategic thinking and their financial management among other organizational practices remains under-researched [16]. To further explain, NGOs are heavily reliant on foreign funding, and therefore, a lack of diverse funding sources and skilled human resources, due to competition from the for-profit sector, hinders their ability to execute programs and adapt creatively to changing needs [17]. From a managerial perspective, bureaucratic red tape and administrative hurdles divert resources from their primary missions, while lack of coordination leads to inefficiencies and gaps in service delivery [18]. Mainly, NGOs often prioritize immediate needs over long-term planning due to unstable environments, impeding the development of sustainable solutions [19]. Due to the significant social responsibilities NGOs bear and the heavy burden they face in providing humanitarian services, often driven by interventions from donors and other stakeholders, achieving sustainability can become a less of an important priority that is challenging to attain. Therefore, this study aims to uncover the key factors and enablers—particularly strategic planning, managerial capabilities, and financial management—that empower NGOs to survive and thrive in crisis-laden environments, even amid resource shortages and scarcity. By focusing on these critical elements, the research seeks to provide actionable insights to enhance the resilience and long-term sustainability of NGOs operating under extreme pressures.

A survey was conducted with 75 NGO professionals in Lebanon, a particularly relevant context, where NGOs face unique and significant challenges. Lebanon’s complex socio-political landscape, economic instability, and frequent humanitarian crises create a highly demanding environment for NGOs. These burdens make it exceptionally difficult for NGOs to achieve sustainability, underscoring the importance of robust strategic planning, sound financial management, and distinctive managerial capabilities. The study will integrate RBV and Stakeholder Theory to create a comprehensive approach to managing resources and relationships both internally and externally. [20] discuss how Stakeholder Theory complements the RBV by adding perspectives on sustainability, normativity, and cooperation, which are essential for NGOs operating in resource-constrained environments. This incites on exploring how NGOs can navigate their unique challenges and capitalize on their strategic resources and stakeholder relationships to achieve a sustainable success [20,21].

The rest of this paper is structured as follows: Section 2 reviews the relevant literature on NGO sustainability in its definition followed in this study, strategic planning, managing capabilities, and financial management. Section 3 outlines the quantitative method used to collect and analyze data. Section 4 presents the findings and discusses their implications for NGO sustainability in Lebanon. Finally, Section 5 concludes the paper by summarizing the key insights and offering recommendations for policymakers, donors, and NGOs’ practitioners.

## 2. Literature review

### 2.1 NGOs’ sustainability and resilience

NGOs’ continuity has emerged as a central idea, with strategic practices becoming a need for NGOs in a dynamic and complex environment. Despite the major role of NGOs in development, the issue of local NGOs’ sustainability remains a major concern in many developing countries [22]. That is, the ability or capacity of the NGOs to endure while considering all the internal and external elements that affect their operations and survival [23,24]. However, by adopting strategic practices, NGOs are better equipped to overcome adversity and leave a lasting impact on society [25].

[26] found that NGOs use new and different languages to illustrate their value, their sustainability, and their resilience as emergent terms in the sector. The term sustainability for instance, has been used as an objective at the organizational level. When sustainability is applied to the operations of organizations, it is considered to be “the ability of administrators to maintain an organization over the long term” [27,28] in their turn, define a sustainable NGO as an organization that can continue to fulfill its mission with time while taking into consideration all the needs of its major stakeholders, particularly its beneficiaries and supporters.

Related to sustainability, increasingly the term resilience that has its origin in ecological science, is appearing in the NGO sector. Despite how debatable this term seems to be in literature, it actually continues to develop on how to define, measure and test for resilience [29]. Resilience can be defined as “the ability to recover from setbacks, adapt well to change, and keep going in the face of adversity” [30]. The literature frequently describes the NGO sector as resilient, implying that NGOs carry the responsibility of adapting to challenging circumstances. However, this emphasis on resilience may limit the establishment of political and policy frameworks that could proactively prevent social issues and instability [26]. Consequently, stressing resilience shifts the focus onto individuals, NGOs, and communities, compelling them to continuously respond and adapt to uncertainty and adversity.

Mission continuity is the bedrock principle of sustainability, with NGOs needing to ensure their activities endure despite challenges [31]. Adaptive techniques that enable NGOs to adjust effectively to changing circumstances while remaining resolutely aligned with their primary mission are essential for creating a durable and resilient organization. An NGO must be financially secure and operationally reliable to effectively address society’s most urgent problems, benefiting both individuals and communities [31]. Successful NGOs work hard to build strong relationships with those they aim to help, fostering resilience and adaptability. Involving all relevant parties in strategic planning enhances community support and collaboration [3]. Active participation from the community increases NGO’s credibility and extends its influence [4]. A flexible and well-informed staff is also crucial for sustainability, requiring substantial commitment to internal learning and development [32]. Investing in human capital boosts efficiency, encourages creativity, and positions the NGO at the forefront of best practices [33]. By emphasizing internal learning, NGOs ensure their staff can execute current projects effectively and respond to new developments [34].

This study conducts a comprehensive analysis of how effective managerial and financial strategies for NGOs can lead to its sustainability over time, providing empirical evidence on the best approaches for Lebanon’s settings. Additionally, the study will investigate tailored capacity-building practices, offering insights into how NGOs of different sizes and types can invest in human capital to enhance sustainability. By addressing these gaps, the study will contribute to a more nuanced and practical understanding of sustainability in the NGO sector.

### 2.2 Stakeholder theory

Stakeholder theory is a prominent management framework emphasizing the consideration of all relevant parties, not just shareholders, in business decision-making [32]. This theory posits that organizations are enmeshed in a network of stakeholders, including employees, customers, suppliers, communities, governments, and investors. Essentially, anyone who affects or is affected by the business is considered to be a stakeholder [35]. Stakeholder theory is an organization theory that inherently includes moral meaning. What is crucial about moral meaning is that it can be perceived in the nature of the relationship between the organization and its different stakeholders and managers as a central relationship or central contract between the organization and its stakeholders [36]. Recent work emphasizes empathy and solidarity as core values that connect organizational strategy with the Sustainable Development Goals, underscoring that sustainable management requires ethical commitments and relational practices alongside stakeholder engagement [37].

Stakeholder theory in NGOs emphasizes that an NGO’s success and sustainability depend on effectively managing relationships with a wide range of stakeholders. Stakeholders include anyone affected by or having influence on the NGO’s activities, such as donors, beneficiaries, employees, volunteers, government entities, partner organizations, and local communities. However, stakeholder theory often falls short in addressing the specific needs of NGOs, particularly regarding their sustainability and continuity. It lacks a focus on the unique challenges that NGOs face in achieving long-term viability. This study aims to fill this gap by exploring the factors that contribute to the sustainability of NGOs.

The theory’s emphasis on balancing the interests of multiple stakeholders aligns with sustainability principles, which prioritize long-term value creation and ethical conduct [34]. By considering the interdependence between organizations and their external environments, stakeholder theory supports the notion that addressing stakeholder concerns can build trust and improve community standing [38].

This study will contribute to bridging the gap in stakeholder theory by focusing on critical factors that enhance NGO sustainability. By examining how NGOs can better manage their resources, engage stakeholders effectively, and navigate regulatory and environmental challenges, the study aims to provide a comprehensive framework for NGOs to achieve long-term sustainability, thus addressing a significant gap in stakeholder theory within the NGO context.

### 2.3 Resource-based view theory (RBV)

The RBV theory is a strategic management paradigm that emphasizes leveraging an organization’s unique assets and strengths to maintain a long-term competitive advantage. According to RBV, organizations possess distinct resources that, when effectively managed, help them stand out, generate value, and ensure their longevity [39]. A key tenet of RBV is that not all resources are equal. Some resources are rare, valuable, inimitable, and non-substitutable, making them core competencies that provide a competitive edge [40]. These resources can include organizational culture, stakeholder relationships, intellectual property, human capital, and physical assets. RBV advocates for resource allocation decisions based on a company’s specific strengths and weaknesses. By aligning resource allocation with competitive advantages, organizations can enhance their chances of thriving and sustaining their operations [41]. Investing in the development of these unique assets allows companies to differentiate themselves in the market, boosting profitability and longevity [2].

RBV also recognizes that resources evolve over time. Organizations must adapt to shifting market conditions, new opportunities, and changing stakeholder needs. Emerging and disruptive technologies such as artificial intelligence, blockchain, and the Internet of Things are increasingly recognized as accelerators of sustainability, enabling NGOs and other organizations to enhance transparency, efficiency, and innovation in resource deployment [42].

Success in a dynamic environment requires flexible and responsive resource allocation strategies [3]. RBV encourages a forward-looking approach to strategic decision-making, ensuring organizations invest in and develop resources that provide a sustained competitive edge, even in volatile markets [4]. However, in the context of NGOs, RBV often overlooks the unique challenges these organizations face in achieving sustainability. This study aims to address this gap by exploring how NGOs can leverage their distinctive resources to enhance their long-term viability. By focusing on the strategic management of resources specific to NGOs, this study will contribute to bridging the gap in RBV theory, providing a framework for NGOs to achieve sustained success and impact.

### 2.4 Variables’ conceptualization: strategic planning, management capabilities, and financial management

Stakeholder theory and the RBV provide a framework for assessing and improving non-profits’ long-term viability. Stakeholder theory promotes a more inclusive strategy for sustainability by considering a wide range of social factors, while RBV encourages non-profits to concentrate on their internal strengths. Together, these theories argue that the long-term success of non-profits relies on a holistic strategy that balances external ties and internal strengths.

Many NGOs in Lebanon rely heavily on funding from foreign donors, foundations, and government agencies. This dependency makes them vulnerable to economic downturns and changes in donor priorities, complicating future planning. Therefore, strategic planning is crucial for overcoming obstacles and seizing opportunities, particularly in volatile, hostile, and dynamic environments [43]. Strategic planning helps NGOs create an overarching goal that advances their purpose over time, serving as a compass for decision-making and maintaining focus on their mission [44]. In Lebanon’s volatile political and social climate, strategic planning enables NGOs to adapt to changing conditions and seize new opportunities, ensuring effective service to their communities. Limited funding is a typical issue for NGOs; therefore, strategic planning helps allocate resources effectively, increasing the impact of limited funding by prioritizing high-impact programs [45].

NGOs in Lebanon also face obstacles that require competent management and solid financial practices. Effective leadership and transparent governance are critical for building constituent trust and ensuring resource stewardship [46,47]. Effective stakeholder involvement fosters collaboration, support, and shared responsibility for the NGO’s mission [32]. Moreover, sound financial management practices, including budgeting, transparency, and diverse funding sources, are essential for economic stability.

Investing in the growth of staff and volunteers increases organizational efficiency. Thus, training, professional development, and a learning culture ensure NGOs can adapt to new situations. Strategic planning, managerial talents, and responsible financial management are crucial to the long-term success of Lebanon’s NGOs, helping them navigate the country’s complexities and emergencies [48] (Table 1).

**Table 1:** Studies on NGOs’ Sustainability Enablers.

Main Studies	Intend to Measure
[32,48–50]	Strategic planning practices and organizational skills that enhance NGO success, long-term sustainability, and adaptability in volatile environments.
[7,33,51,52]	Leadership effectiveness, managerial capabilities, governance structures, stakeholder engagement, and human resource capacity as enablers of organizational sustainability.
[53–56]	Determinants of NGO financial performance, including budgeting, reporting, income diversification, financial controls, and long-term financial resilience.

A notable study in the area of NGOs sustainability in Lebanon is [49] that emphasizes the importance of strategic management, financial management, and managerial skills in Lebanese NGO’s sustainability. It highlights the need for skilled project managers and governors in NGOs to provide clear directions and diversify funding sources. Similarly, [57] emphasize the importance of financial sustainability and adequate remuneration for skilled staff in South African NGOs. [58] discusses the link between strategic management systems and sustainable business models, emphasizing the integration of sustainability and financial success. RBV has been widely applied in strategic management across sectors, including NGOs, where organizational culture, networks, and knowledge resources serve as inimitable assets [2,7]. This underscores RBV’s relevance beyond private firms to mission-driven organizations, while also benefiting from integration with stakeholder theory, which can provide normativity, sustainability, and cooperation to inform RBV [20]. Within the NGO context, stakeholder theory emphasizes that sustainability depends on managing diverse relationships with donors, beneficiaries, governments, and communities. Empirical studies show that NGOs’ ability to adapt and remain accountable is directly linked to inclusive stakeholder engagement [22], and that donor dependency shapes responsiveness by influencing governance and sustainability [59]. More recently, [1] highlight that NGOs balancing competing stakeholder demands are more likely to achieve long-term viability, while [34] caution that generic applications of stakeholder theory risk overlooking the unique institutional challenges NGOs face. Taken together, research on sustainability management in NGOs points to the significance of combining top-down strategic direction with bottom-up personal initiative, illustrating how these dynamics align with the principles of both RBV and stakeholder theories [60].

#### 2.4.1 Strategic planning.

Strategic planning provides a systematic framework for guiding an organization’s decisions and coordinating activities [43]. For NGOs, strategic planning formalizes the mission and vision, helping maintain focus and adapt to changing conditions. By anticipating and planning for alternative outcomes, NGOs can navigate volatility with flexibility and resilience [43]. Strategic planning enables efficient resource allocation by prioritizing critical initiatives, increasing the impact of limited funds [45]. It also encourages stakeholder participation, fostering a sense of ownership and support [46]. Implementing strategic planning at all organizational levels promotes collaboration, productivity, and innovation, addressing complex problems and achieving long-term success [34,35]. It was discovered by [61] that NGOs typically leverage strategic management systems and perceive strategic management as a crucial tool for securing a top quality of service delivery, as well as achieving goals and increasing overall performance.

Miles and Snow, among others, argue that strategy content is an important aspect that leads to better organizational performance. Evidence from Vietnamese SMEs confirms that strategic management practices foster organizational innovation, which in turn strengthens financial performance [62]. This suggests that strategy formulation and implementation are critical drivers of competitiveness and long-term sustainability. Also, results from [63], show that strategy can be separated out from other elements of management for a distinguishable assessment of its impact on the overall organizational performance and continuity. It’s expected from NGOs to leverage strategic planning tools to transform the public funds and donations into value-added services [50] especially since it’s seen to have a significant influence on the financial sustainability of NGOs [64]. Ultimately, the first hypothesis would be:

H1: Strategic planning has a positive direct influence on NGOs sustainability.

#### 2.4.2 Management capabilities.

Competent administration is essential for NGO success and longevity. Effective leadership provides direction and motivates stakeholders towards common goals [33]. Strong stakeholder relationships, efficient operations, and adaptability are crucial management capabilities. Skilled management fosters collaboration, partnerships, and a healthy organizational culture, enabling NGOs to tackle social issues effectively [35,38].

Retention practices that recognize and reward employee achievements enhance morale, job satisfaction, and organizational commitment, ensuring the continued provision of services and successful strategic implementation [65]. Job involvement that is due to the support received from management is proven to increase employees’ in-role performance and positively influences their extra-role behavior as well [66]. In a study conducted by [51], all the four dimensions of transformational leadership have been found to have a positive effect on organizational performance where also employee satisfaction played a mediating role.

Many empirical studies indicate that solid management capabilities are positively and directly related to better organizational performance and long-term sustainability. For instance, a study in Jordan found that local NGOs with higher capacity in governance and human resources tend to perform significantly better [52]. Also, in a different study conducted for Nepal’s IT sector, it has been found that firms that develop their ability to sense, adapt to environmental changes, and reconfigure their resources, which are often known as dynamic capabilities, do show stronger resilience, which in turn leads to improved performance [67]. Research in the public sector of the UAE also shows that organizations that invest in preparedness, risk management, and support from leadership for sustainability practices tend to maintain higher levels of performance with time [68]. Furthermore, NGOs in Jordan were able to increase their competitiveness and learning when they enhanced their internal resources, experience, and ability to share knowledge among staff [69]. The second hypothesis suggests:

H2: Managerial skills and capabilities have a positive direct influence on NGOs sustainability.

#### 2.4.3 Financial management.

Effective financial management boosts an NGO’s social impact and long-term viability. It involves prudent resource allocation, transparency, and risk mitigation strategies to ensure stability [41]. Financial viability allows NGOs to weather economic fluctuations and sustains operations. Transparent financial practices build donor confidence and stakeholder trust, essential for continued support [40].

Financial management also informs long-term strategy through data-driven decision-making, optimizing resource use and enhancing social impact. By maintaining financial stability and preparing for future challenges, NGOs can fulfill their missions and amplify their social effects [70,71].

Sound financial management is widely recognized as a key determinant of NGOs’ performance, sustainability, and continuity. For example, [56] argues that in NGOs, sound financial planning, budgeting, reporting, and internal controls are essential for achieving organizational goals and maintaining donor trust; without such practices, NGOs often struggle to deliver services efficiently and to sustain operations over time. In a case study of YMCA Ghana, [54] found that indicators such as the quality and responsibility of financial management staff, the degree of financial control and reporting, and effective budget management significantly correlate with better performance and longer organizational survival. In Kenya, a recent empirical study in the Lower Eastern Counties demonstrated that budget control, cash management, income diversification, and financial monitoring positively influence financial performance of NGOs, thus reinforcing their resilience and continuity [55]. Finally, research in Portugal among non‐profit associations showed that human resource management, especially through trained, motivated staff, and strategic financial practices are instrumental in sustaining financial outcomes, even in contexts of economic instability, thereby ensuring continuity of mission [53]. Thus, the third Hypothesis posits:

H3: Robust financial management has a positive direct influence on NGOs sustainability.

### 2.5 Conceptual framework

**Fig 1:**
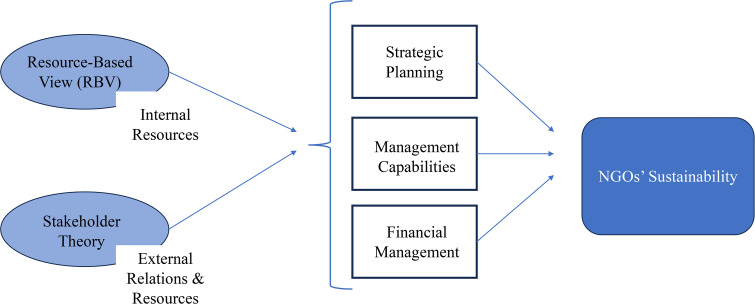
Conceptual Framework.

In the literature review, the inquiry begins with the theoretical underpinnings of our research into the interconnectedness of NGO sustainability, the (RBV), and stakeholder theory through the three enablers: strategic planning, managerial competence, and financial management. The conceptual framework in Fig 1 depicts the interplay between these factors.

The diversity of perspectives on sustainability, enriched by Stakeholder Theory and the RBV, offers a multifaceted analysis, especially for Lebanese NGOs. In addition to internal resources and stakeholder dynamics, recent research emphasizes that sustainability outcomes are also shaped by the broader institutional and economic environment. [72] demonstrate that environmental and organizational sustainability depends not only on institutional effectiveness but also on economic capacity, reinforcing the view that NGOs in fragile contexts operate under systemic constraints that internal strategies alone cannot overcome. This highlights the need to situate NGO sustainability within multi-level dynamics that combine internal capabilities, stakeholder relations, and institutional–economic determinants. These theories help examine the interplay of strategic planning, managerial practices, financial policy, and enduring success.

## 3. Methods

### 3.1 The context

Lebanon has a complicated social and political climate owing to its history of conflict, economic inequality, and regional tensions [41]. In the absence of the governmental support, whether this concerns the weak governance and the political instability, or the inefficiency and corruption in public services delivery, or even the economic constraints that have existed for several years and the limited government response to these crises, NGOs play a crucial role in helping Lebanon address its myriad social, economic, and environmental issues. They are vital in tackling service delivery disparities, advocating for human rights, and promoting sustainable development [2].

In fact, Lebanon’s history has been indelibly shaped by its geopolitical tensions and the devastating civil war. Indeed, Lebanon’s NGOs face significant challenges impacting their long-term sustainability and effectiveness in addressing critical social, economic, and environmental issues. The sector operates within a complex sociopolitical landscape marked by past conflicts and ongoing regional tensions, making reliable operation difficult. Economic instability, characterized by high inflation and unemployment, further exacerbates their operational challenges, often reducing donor support and financial stability [18,73,74].

For instance, as Lebanon’s economy spirals into one of the worst crises in modern history, many NGOs have struggled to maintain funding levels, forcing them to cut back on services or even shut down operations entirely [75,76]. Moreover, the Lebanese government’s failure to provide adequate support during these crises has placed additional pressure on NGOs to fill the gaps left by the state, stretching their resources thin [77]. The ongoing economic hardships have also led to a reduction in international donations, as global donors become more hesitant to invest in a country with such uncertain prospects [78].

### 3.2 Research design

Two main paradigms are commonly used in research: the positivist research and the interpretivist critical research [79]. The former is linked to the quantitative methods for it provides the science an objective view; whereby, deductive reasoning is applied to operationalize the chosen theories into a quantitative framework. In comparison to previous research (e.g., [49,53]; Abu [52] etc.) on NGO sustainability, this study adopts a more comprehensive and rigorous methodological approach. Former studies primarily employed survey-based data collection and regression analysis, focusing on small or convenience samples. While these studies provided important insights, they often lacked triangulation and extensive validity testing. Similarly, other studies examined NGO sustainability through quantitative surveys but reported limited detail on psychometric properties and control variables.

By contrast, our study integrated qualitative and quantitative elements, beginning with focus group discussions to enrich contextual understanding, followed by a structured survey distributed to 75 NGOs operating in Lebanon. This mixed-method design enhances validity by capturing both lived experiences and generalizable patterns. Additionally, the survey instruments were adapted from established measures and subjected to rigorous psychometric testing which confirmed construct validity and internal reliability. Hierarchical regression was conducted with relevant control variables (e.g.,: firm age, respondent age). Assumptions of regression analysis were also thoroughly checked and met.

Therefore, compared with similar studies in the field, our methodology demonstrates stronger robustness through its multi-stage design, comprehensive validity checks, and contextual application in Lebanon’s highly volatile environment, which provides a rigorous stress test for the proposed framework. Hypotheses are formulated based on the theoretical constructs of the Stakeholder Theory and the RBV, positing that strategic planning, managerial skills, and financial management are predictors of NGO sustainability in Lebanon. These hypotheses are then subjected to empirical testing through the collection and analysis of quantitative data.

### 3.3. Data collection and analysis

The sample for this study was determined through a non-probability sampling technique, considering the feasibility and resources available. Before the distribution of the questionnaires via Google Forms that started in July 10, 2023 and lasted till October 3, 2023, and to adhere to ethical research practices, a consent form was required to be signed by the respondents. Both the consent form and the questionnaire have been approved by the University’s Institutional Review Board (IRB) which certainly strengthens the integrity of the research process.

Regarding instrumentation, the measures employed in the survey are grounded in the scholarly work of previous researchers, including [80,81], and the survey developed by [82]. Some of the measurements were slightly adjusted to suit the context of the study. Psychometric tests were further conducted to ensure the validity and reliability of the instruments (e.g., Table 2). The survey constitutes of 31 questions and was administered to decision-makers within NGOs operating in Lebanon. Questionnaires were distributed to 100 NGOs, chosen based on their availability and willingness to participate. Out of the distributed surveys, 75 agreed to participate in the study, providing a robust and sufficient sample for comprehensive analysis. The survey data has been accessed for research purposes on October 5, 2023.

**Table 2:** Descriptive Statistics for Strategic Planning (SP), Management Capabilities (MC), Financial Management (FM), and Sustainability (S).

Variables	Mean	Median	SD	Min	Max
Strategic planning	3.75	4	0.67	1.86	5
Management Capabilities	3.65	3.77	0.68	1.78	5
Financial Management	3.62	3.66	0.73	2	5
NGO’s sustainability	3.52	3.6	0.75	1	5

A three-step hierarchical multiple regression was conducted with sustainability being the dependent variable, strategic planning as predictor was entered as the first step, followed by managerial skills and financial management as the last step. SPSS version 25 was used for conducting Pearson correlation tests and regression analysis where the total R squared value after each step was generated, while observing variations in R squared and the significance of change in predictability. A number of assumptions has to be met for conducting the hierarchical regression analysis. Initially, all predictors which had a tendency to be significant in correlation tests (P < 0.1) were considered for the regression analysis. Results also showed the absence of multicollinearity for conducting a proper regression analysis, where the predictors in the model had a correlation less than 0.8, with VIF values <10. Other assumptions for conducting regression analysis were also checked, such as inspecting the scatterplot that showed no outliers with a standardized residuals greater than 3.3 or less than −3.3, normality of the dependent variable, linearity and homoscedasticity, hence, satisfying the assumptions for regression analysis.

To operationalize the constructs of strategic planning, management capabilities, and financial management, we designed a survey instrument based on established NGO and strategic management literature. Each measurement item was selected for its theoretical relevance and empirical validation and later confirmed through Principal Component Analysis (PCA), which grouped them into coherent factors reflecting NGO practices.

Strategic planning was measured through items assessing (1) mission clarity, (2) stakeholder involvement in planning, and (3) alignment of long-term goals. Mission clarity captures the degree to which NGOs maintain a consistent and shared purpose, a recognized determinant of sustainability [4]. Stakeholder involvement reflects the participatory nature of planning processes, which enhances legitimacy and adaptability in volatile environments [1]. Long-term goal alignment indicates strategic foresight, essential for sustaining operations amid uncertainty [28]. PCA results confirmed that these items loaded strongly on the Integrated VP Practice factor, indicating that clarity and alignment are embedded in holistic organizational practice.

Management capabilities were assessed through (1) leadership effectiveness, (2) staff coordination, and (3) transparency in decision-making. Leadership effectiveness reflects the ability of NGO leaders to mobilize resources, motivate staff, and adapt to crisis conditions [83]. Staff coordination ensures efficient operations, particularly under resource constraints [59]. Transparency fosters trust and accountability, which are critical for donor relations and stakeholder confidence [22]. These items aligned closely with the Customer-Driven Value Foundation factor in the PCA, underscoring how internal managerial practices reinforce external responsiveness to beneficiaries and donors.

Financial management was measured through (1) budget discipline, (2) diversification of funding sources, and (3) compliance with donor reporting. Budget discipline reflects the NGO’s ability to plan and control expenditures under conditions of economic volatility [57]. Funding diversification reduces dependency risks and enhances resilience to donor withdrawal [49]. Donor reporting compliance represents accountability mechanisms that maintain credibility and ensure future funding [84]. Interestingly, while these items formed a coherent scale, the PCA revealed weaker loadings on performance-oriented factors, reflecting the Performance Accountability Gaps observed in many Lebanese NGOs. This suggests that while financial controls exist, they may not consistently translate into measurable sustainability outcomes under crisis conditions.

### 3.4. Descriptive

#### Profile of participating NGOs.

The 75 NGOs that participated in this study represent a diverse cross-section of Lebanon’s non-profit sector. Approximately, 40% were small to medium-sized organizations with fewer than 500 employees, while 2% were larger entities employing more than 500 staff. In terms of years of operation, 39% had been operating for less than seven years, while the other 61% had been operating for more than 7 years indicating substantial experience in project implementation and donor relations. The NGOs’ areas of focus included in its most, community development and social services accounting for almost 51% of the surveyed NGOs, Humanitarian and Emergency Relief 12%, education 11%, health and medical services 9%, Child Protection and Child rights 8%, and many more. This diversity enhances the representativeness of the data and strengthens the applicability of findings across Lebanon’s NGO ecosystem.

#### Profile of respondents.

Table 3 presents the descriptive statistics of the demographic information for the participants. From the total of 75 participants, 46 (61.3%) were female, 26 (34.7) were male, and 3 (4%) preferred not to disclose their gender. The majority of the participants (n = 69, 92%) were above the age of 25 years. Similarly, the vast number of participants had either a bachelor or Master’s degree (n = 66, 88%) with only one participant with a secondary school diploma. The participants had different occupations, most frequently work related to programs/projects (n = 36, 48%), then general management (n = 22, 29.3%), followed by finance and administration (n = 10, 13.3%) and communication/PR (n = 7, 9.3%).

**Table 3:** Descriptive Demographic Statistics.

Factors	Frequency	Percentage
*Gender*		
Female	46	61.3
Male	26	34.7
Prefer not to say	3	4
*Age*		
>36	31	41.3
18-25	6	8
26-35	38	50.7
*Level of Education*		
Bachelor/Master	66	88
Higher vocational	3	4
Ph.D./DBA	5	6.7
Secondary school	1	1.3
*Working Department*		
Communication/PR	7	9.3
Finance & Administration	10	13.3
General Management	22	29.3
Programs/projects	36	48
*NGOs Years of Operations*		
< 1 year	3	4
> 7 years	43	57.3
1-3 years	9	12
3-7 years	20	26.7

### 3.5. Principal component analysis

#### Reliability and validity.

Prior to survey administration, three virtual focus groups, each involving 8–10 NGO representatives, were conducted to refine the wording and contextual relevance of measurement items. The qualitative insights derived from these sessions ensured that the constructs reflected the lived realities of Lebanese NGOs, thereby strengthening content and face validity. The study’s conceptual model and measurement domains were grounded in prior research that established their theoretical relevance to NGO sustainability. While these earlier works guided construct selection conceptually, the present study independently verified their empirical robustness through multiple statistical tests.

Specifically, construct validity was confirmed via Principal Component Analysis (PCA), which yielded factor loadings above 0.4 and clear component separation for all four constructs: strategic planning, managerial capabilities, financial management, and sustainability. Reliability was demonstrated through high internal consistency, with Cronbach’s alpha coefficients exceeding 0.70 (SP = 0.88; MC = 0.90; FM = 0.90; S = 0.89). Convergent validity was evidenced by significant inter-item correlations, and discriminant validity was established as inter-construct correlations remained below 0.80. In addition, expert review by three academic scholars and two senior NGO practitioners further confirmed the adequacy and clarity of all survey items. Collectively, these procedures provide strong evidence that the study’s constructs are both reliable and valid for examining NGO sustainability in Lebanon’s context.

PCA was conducted to assess the psychometric properties of the questionnaire that entailed 24 items (questions) that measure the variables of the research framework (SP, MC, FM, and sustainability). PCA determines whether the observations are valid and reliable. The first step involved assessing the suitability of the data for PCA though Keiser-Meyer-Olkin (KMO) and Barlett’s test of sphericity (BTS) that showed significant results (KMO: 0.87 > 0.5; BTS: X = 1622, df = 351, P < 0.01) [85]. In addition, the correlation matrix showed numerous variables to be correlated (>0.3) which confirms the suitability of the data. In the second step, factor analysis was conducted to assess whether the items related to each of the four components (SP, MC, FM, S) load together with a factor loading > 0.4. The results shown in Table 4 verify this assumption, with 7 items for SP, 6 items for MC, 6 items for FM, and 5 items for sustainability. The third step was to assess the internal consistency reliability of each of the four components using Cronbach’s alpha that should surpass the value of 0.7. The results showed satisfactory Cronbach’s alpha values for SP (α = 0.88), MC (α = 0.9), FM (α = 0.9), and sustainability (α = 0.89).

**Table 4:** Factor Loadings For SP, MC, FM, and S.

Items	Factor Loading	
Stakeholders’ contribution to NGO’s programs and projects	.634	SP
Sharing organization’s mission	.496	
Alignment of NGO’s goals, vision, and mission	.670	
Sound and comprehensive strategic assumptions	.736	
Adoption of strategic planning	.643	
Strategic planning provides guidance to fulfil mission and goals	.654	
Changes and adaptation of strategic plans	.491	
Awareness of top management and effective leadership	.673	MC
Implementation of procedures and policies for employee retention	.808	
Leadership effectiveness towards achieving goals	.546	
Ability to acquire stakeholders’ trust	.502	
Effective decision-making	.467	
Skilled, involved, and professional staff	.689	
Financial policies to manage its funds.	.643	FM
Effective leadership for attractive donors	.695	
Clear and organized reporting structure for accountability and openness	.768	
Solid brand name that appeals to donors	.687	
Solving financial problems	.796	
Adequate finances	.689	
Strategic plan for long-term sustainability	.763	S
Diversification of funding sources	.796	
Capacity for internal learning and development	.634	
Program adjustments for target beneficiaries	.664	
High predictability and consistency of funding	.699	

*A shortened form for each item is displayed.

### 3.6. Pearson correlation and hierarchical regression analysis

Pearson Product Moment correlation was conducted as a preliminary step for hierarchical regression analysis to examine the strength and direction of linear relationships between pairs of continuous variables (SP, MC, FM, Sustainability) and to ensure the linearity and independence of the variables to be inserted in the regression model.

The results show strategic Planning having a strong significant positive correlation with Sustainability (r = 0.759, p < 0.01), indicating that organizations with more robust strategic planning processes tend to exhibit higher levels of sustainability. Similarly, Management Capabilities demonstrates a strong positive correlation with Sustainability (r = 0.702, p < 0.01), implying that effective leadership, competent decision-making, and skillful management of resources play a significant role in ensuring the sustainability of NGOs. In addition, financial Management also exhibited a moderate positive correlation with Sustainability (r = 0.636, p < 0.01), suggesting the importance of sound financial planning, budgeting, and resource allocation in maintaining the long-term viability of NGOs.

We employed hierarchical regression to test our three hypotheses using version 25.0 of SPSS. The independent variables of the hypotheses were introduced sequentially, resulting in three models shown in Table 5. This study included two control variables – age of the respondent and firm age – given that these variables can influence sustainability levels [86–88] (Table 6).

**Table 5:** Pearson’s product moment correlation between SP, MC, FM, and S.

	1	2	3	4
SP (1)	1			
MC (2)	.782^**^	1		
FM (3)	.665^**^	.712^**^	1	
Sustainability (4)	.759^**^	.702^**^	.636^**^	1

**Table 6:** Hierarchical Regression Models between SP, MC, FM and Sustainability.

	Sustainability		
	Model 1	Model 2	Model 3
Control			
Respondent Age	−.92 (.13)	−.58 (.35)	−.65 (.12)
Firm Age	.11 (.126)	.1 (.122)	.11 (.12)
Variables			
H1: SP	.76** (.09)	.54** (.14)	.5** (.14)
H2: MC		.26* (.13)	.18(.14)
H3:FM			.17(.11)
F	34	28	23
R2	.59	.61	.63
Adj. R2	.57	.60	.61
ΔR2	.53	.027	.014
Df	74	74	74
P	<0.01	<0.01	<0.01

Concerning H1, SP showed a significant positive association with sustainability in model 1 (β = 0.76, p < 0.01), being the first predictor introduced in the hierarchical regression. In addition, SP also showed same significant results in model 2 (β = .54, p < 0.01), and model 3 (β = .29, p < 0.01) with the introduction of other variables. This shows sufficient evidence to support H1. Model 1 is shown to be statistically significant F (3, 71) = 34.01, p < 0.01], with SP being a strong predictor for sustainability, counting for a 57% variance (R square = 0.154; Sig F change <0.01). To test for H2, MC was introduced in the second model which slightly increases the variance in sustainability by 3% (β = .26, p < 0.01, Sig F change <0.05), counting for a total of 60% variance while model 2 as a whole being statistically significant [F (4, 70) = 28.11, p < 0.01]. Therefore, it can be argued that there is evidence to support H2, however, MC has a weak role in enhancing sustainability. In regards to H3, Model 3 shows FM to have no significant contribution to the prediction of sustainability (β = .17, p > 0.05) despite SP slightly increasing the variance in sustainability by 1% [F (5, 69) = 23.6, p < 0.01]. Therefore, there is not enough evidence to support H3.

## 4. Discussion

The objective of this paper is to show whether management capabilities, strategic planning, and financial management can actually help in achieving sustainability in a very difficult context where NGOs’ survivability is actually dependent on fund givers. Also, the objective elaborates to explore whether internal processes of these NGOs are fit to achieve sustainability, but most importantly if sustainability as an objective is on their agenda while operating in this specific context. By integrating both the stakeholder theory and the RBV, it’s shown that a combination of internal and external resources that the NGO holds can make an impact in how long this NGO sustains and persists. Currently, much of the existing literature on this topic have not explored it linking these resources together and testing their immediate relationship with sustainability.

The statistical results from the study indicate a significant positive relationship between strategic planning and organizational sustainability, highlighting the importance of strategic foresight in ensuring enduring success. This has been emphasized and argued in previous research by [89] and [43] that depicted the importance of aligning goals with the organization’s mission and focusing on long-term results, which makes strategic planning create a lasting positive impact. Indeed, an NGO’s mission, vision, goals, and objectives may be clearly defined via strategic planning, which also involves developing tactics to successfully reach objectives. Strategic planning enables businesses to foresee and adapt effectively to changes by considering alternative outcomes and developing backup plans. This adaptability, supported by well-planned plans, gives NGOs the strength and agility to deal with the unexpected.

Our results resonate with [35,42], and [90], who argue that integrating sustainability into strategic planning enhances stakeholder trust, fosters employee collaboration, and improves organizational resilience by addressing challenges such as resource depletion, climate change, and social inequities. Similarly, our finding that strategic planning significantly enhances NGO continuity aligns with [22,25], and [28] who emphasized accountability, mission clarity, and resilience. Notably, in Lebanon’s highly volatile context, strategic planning retains predictive power even under unstable financial conditions, extending prior conclusions to fragile state environments Now, in regards to the association of management capabilities and sustainability, the findings of this study emphasize the foundational role of those capabilities in fostering long-term performance and sustainability of NGOs. Effective leadership is crucial in this relationship, as it enables strategic decision-making that aligns with the broader organizational vision. Leaders who can foresee the bigger picture and make calculated strategic choices are instrumental in guiding their organizations towards sustained success.

On this note, [91] emphasized the role that managerial skills play in helping organizations create resilient cultures that can adapt to changes and uncertainties, which enhances organization’s sustainability. This goes in line with the results received during this study that argue the cruciality of managerial skills in streamlining operations. Organizations with strong management practices are better equipped to withstand challenges and adapt to new situations. The results that validated the second hypothesis show that management capabilities in its different aspects does impact NGO’s sustainability which highlights on fostering an environment that encourages open communication and collaboration within the organization. [92] advocated this perspective mentioning that this culture of resilience ensures that organizations can navigate through crises and emerge stronger.

The level of stakeholder engagement affects NGOs’ performance, managerial skills, and longevity. The goal of stakeholder-centric management is to prioritize the needs of all stakeholders, including employees, volunteers, beneficiaries, donors, the government, and local communities. The connections built via this technique help ensure sustainability by providing mutual support, insightful contributions, and shared ownership of long-term objectives. Stakeholders who are actively interested and engaged become advocates for the sustainability of an organization, which boosts its chances of making a good impact as it was explored by [93]. The positive effect of management capabilities aligns with Arbab Kash et al. (2014), who linked leadership and staff coordination to NGO effectiveness, and with Lewis et al. (2020), who emphasized balancing competing stakeholder demands. Our results also support [17], who found that human resource constraints weaken NGO adaptability. In contrast, [34] warned that generic managerial models overlook NGO-specific challenges; our data reinforce this by showing that Lebanese NGOs rely on locally contextualized managerial skills, which helps explain their continued operation despite systemic crises.

Despite the common assumption that financial management is a significant determinant of NGO sustainability, the findings contradict previous literature and indicate otherwise. This actually opposes the widely held belief that sound financial practices directly enhance an organization’s ability to endure and thrive. In fact, [94] found no empirical evidence linking financial management practices to NGO sustainability. Indeed, other factors, such as stakeholder engagement and community involvement, were identified as more crucial for achieving long-term sustainability [95]. Also, sustainability is perceived to be a muti-dimensional and complex factor. The findings of [96] emphasize the multidimensional nature of sustainability, which is influenced by a variety of elements beyond just financial health The non-significance of financial management diverges from [57], who identified financial controls as decisive for NGO survival in South Africa, and from [84], who emphasized donor reporting compliance as a sustainability driver. The non-significance of financial management in predicting NGO sustainability in Lebanon can be better understood by examining the country’s unique institutional and socio-economic environment. Unlike more stable settings where sound financial practices directly translate into organizational continuity, Lebanese NGOs operate in a fragile context marked by prolonged economic crisis, political instability, hyperinflation, and volatile donor funding streams. Under such conditions, even the most robust financial systems cannot guarantee sustainability, as external shocks and sudden shifts in donor priorities often outweigh internal budgeting or accountability mechanisms.

Moreover, Lebanese NGOs are heavily donor-dependent, with limited opportunities for income diversification or financial autonomy [49]. This dependence diminishes the strategic value of financial management as an internal capability: compliance with donor requirements and short-term financial reporting often take precedence over long-term financial planning. In other words, financial management is reduced to an administrative function rather than a strategic driver of sustainability. Donors may also impose earmarked funding, restricting NGOs’ discretion in resource allocation and weakening the link between internal financial practices and organizational durability.

Another contextual factor relates to the prioritization of immediate service delivery in crisis settings. Lebanese NGOs are frequently compelled to channel resources toward urgent humanitarian needs such as food security, healthcare, refugee assistance, leaving limited scope for long-term financial strategizing. This operational reality aligns with findings from [53,96], who note that in unstable environments, sustainability is shaped less by financial systems and more by managerial adaptability, stakeholder trust, and community embeddedness. Thus, while financial management remains important, in Lebanon’s volatile environment its impact is overshadowed by the stronger influence of strategic foresight and managerial capabilities in enabling NGOs to endure.

Therefore, while financial management is undoubtedly important, its role in predicting NGO sustainability appears limited. Instead, a broader approach encompassing various other factors is essential for achieving sustainable outcomes for NGOs which could be a potential topic for further exploration in future research.

### 4.1. Theoretical contributions

This study contributes to theory by integrating Stakeholder Theory and the Resource-Based View (RBV) in the context of NGO sustainability, offering a more holistic understanding of how external and internal resources jointly influence organizational durability. While prior research has often examined these theories in isolation, our findings demonstrate that sustainability in NGOs cannot be explained adequately by external stakeholder relationships alone, nor by internal resources in isolation. Instead, it emerges from the interaction of both. This combined perspective advances theoretical development by highlighting that stakeholder engagement is not merely an external factor but also a mechanism that strengthens internal capabilities, thereby aligning RBV and Stakeholder Theory in a mutually reinforcing way.

Second, this study extends both theories into an understudied, volatile environment, the Lebanese NGO sector. By testing their explanatory power in a context of prolonged economic and political instability, the study shows that the applicability of these frameworks extends beyond stable markets and into fragile states. This expands the theoretical scope of RBV and Stakeholder Theory, demonstrating their relevance in crisis-laden settings where traditional assumptions about resource stability and stakeholder predictability may not hold.

Third, the study contributes to NGO sustainability literature by challenging the prevailing assumption that financial management is always a core determinant of organizational continuity. Our findings suggest that in volatile contexts, financial management plays a diminished role compared to strategic planning and managerial capabilities. This nuance refines theoretical expectations and points to the need for context-sensitive adaptations of RBV and Stakeholder Theory when applied to non-profit organizations.

In sum, this study advances theory by (1) integrating RBV and Stakeholder Theory into a joint explanatory framework, (2) extending these theories into fragile and crisis-affected environments, and (3) revising assumptions about the weight of financial management in sustainability models. Together, these contributions enrich theoretical debates on how NGOs achieve resilience and continuity in resource-constrained settings.

### 4.2. Practical contributions

From a practical perspective, NGOs should prioritize creating and executing thorough, evidence-based strategic plans that align with their objectives and capabilities. This includes involving all constituencies, whether board members, employees, beneficiaries, or donors, in the planning process to ensure alignment with long-term goals.

While existing theory supports the effect of financial management on NGOs’ sustainability, it came to be proven in this study that practically speaking, financial management needs a multidimensional approach to be considered impactful and in a relationship with sustainability. By this, it’s meant that financial management has to be integrated in a well-set strategic plan and implemented by capable people for it to have the desired results.

Additionally, effective governance is vital for ensuring the sustainability, transparency, and accountability of NGOs. Strong governance frameworks provide the necessary oversight and strategic direction, helping organizations to manage risks, allocate resources efficiently, and maintain stakeholder trust. Investing in capacity-building programs is crucial; NGOs should provide training and mentorship opportunities to develop skills in strategic thinking, decision-making, and execution among their leadership and staff. In addition, NGOs should foster a culture of continuous learning and innovation. Encouraging staff and partners to collaborate, share knowledge, and experiment with new ideas can help identify new opportunities and solutions, enhancing the organization’s ability to respond to emerging challenges.

## 5. Conclusion

This study evaluates the relationship between strategic planning, management capabilities, financial management, and NGO sustainability. The first hypothesis, that establishes a positive correlation between strategic planning and sustainability has been validated. In fact, effective strategic planning aligns organizational missions with broader impacts, fostering adaptability and long-term success by enabling NGOs to navigate complex environments and contribute positively to societal and environmental well-being [94].

The second hypothesis confirms that management capabilities, including leadership, stakeholder engagement, and transparent communication, are essential for organizational sustainability. These capabilities help streamline operations, engage stakeholders, and adapt to changes, ensuring long-term success [83].

However, the third hypothesis, which proposed a positive correlation between financial management and NGO sustainability, was not validated. This suggests that sound financial practices alone are not sufficient to ensure sustainability. Instead, a holistic approach that integrates financial management with strategic planning and effective leadership is necessary for achieving lasting sustainability [96]. Furthermore, a systematic review identified gaps in understanding the complex relationship between financial management and sustainability, suggesting the need for more sophisticated testing [97].

The limitations of this study should be acknowledged to provide a balanced interpretation of the findings. First, the sample size of 75 NGOs, while sufficient for the statistical analyses conducted, remains relatively small and geographically concentrated in Lebanon. This limits the extent to which the results can be generalized across the broader NGO sector, particularly in different socio-political or cultural contexts. Future studies could employ larger and more diverse samples across multiple countries, allowing for cross-national comparisons that reveal whether the relationships observed here hold in both stable and fragile environments.

Second, the use of a purposive, non-probability sampling strategy means that the participating NGOs may not fully represent the heterogeneity of the sector. NGOs with limited resources or those operating in rural or marginalized areas may face different sustainability challenges than those captured in this study. Future research should consider stratified or randomized sampling to ensure broader representation and to capture the perspectives of less visible but equally critical organizations.

Third, this study did not capture all possible dimensions of sustainability. While the focus was on strategic planning, managerial capabilities, and financial management, other dimensions such as governance structures, social legitimacy, community embeddedness, digitalization, and external collaborations were not systematically examined. Future research should adopt a more multidimensional framework, integrating these factors to provide a fuller picture of what drives long-term NGO sustainability.

Finally, only specific aspects of Stakeholder Theory and the Resource-Based View were considered. Future research could build on this by exploring how distinct stakeholder relationships (e.g., with governments, international agencies, or private sector partners) interact with internal capabilities such as innovation, technology adoption, or human resource development. Longitudinal studies would also be valuable to trace how sustainability evolves over time, particularly in crisis-prone environments where NGOs must continuously adapt.

By addressing these limitations, future research can offer a more nuanced and comprehensive understanding of how NGOs achieve resilience and sustainability across diverse contexts. In conclusion, this research highlights the interconnectedness of strategic planning, management capabilities, and financial management in achieving NGO sustainability. While financial management alone may not guarantee sustainability, integrating it with strategic planning and effective leadership can significantly enhance organizational resilience and long-term success, particularly in challenging environments like Lebanon.
